# Inequality of educational opportunity at time of schooling predicts cognitive functioning in later adulthood

**DOI:** 10.1016/j.ssmph.2021.100837

**Published:** 2021-06-05

**Authors:** Anja K. Leist, Eyal Bar-Haim, Louis Chauvel

**Affiliations:** a University of Luxembourg, Department of Social Sciences, Institute for Research on Socio-Economic Inequality (IRSEI), Esch-sur-Alzette, Luxembourg; b Ben-Gurion University of the Negev, Department of Education, Beersheba, Israel

**Keywords:** Social mobility, Inequality of opportunity, Cognitive decline, Longitudinal, Multilevel modeling

## Abstract

**Objectives:**

Our understanding of how societal conditions and educational policies influence cognitive development across the life course is improving. We tested the extent to which inequality of educational opportunity (IEO), the country- and cohort-specific correlation of parents' and their offspring's length of schooling, offers systematically different opportunities to contribute to cognitive development, which in turn influences cognitive abilities up to older ages.

**Methods:**

A total of 46,972 individuals of three cohorts born 1940–63 from 16 European countries and Israel provided up to six cognitive assessments and information on covariates in the SHARE survey 2004–2017. Individual-level data were linked to indicators of IEO at time of schooling, and economic, health, and human development, provided by World Bank, WHO, and the UN.

**Results:**

In multilevel (mixed-effects) models with random individual and country-cohort effects and adjusted for a large set of confounders, higher IEO was associated with lower levels of cognitive functioning in men and women. Interaction analyses suggested lower cognitive levels particularly of women who were schooled in higher IEO contexts and had lower educational attainment. Associations with rate of change in cognitive functioning were present only in women, however there was little clinically relevant cognitive decline across the window of observation. Result patterns were mostly consistent after including additional contextual indicators, and in a subsample with childhood information.

**Discussion:**

Findings suggest that IEO is able to substantially influence cognitive development with long-lasting impacts. Lower-educated women of the cohorts under investigation may have been particularly vulnerable to high-inequality educational contexts.

## Highlights

• Macro-level determinants of older-age cognitive functioning need to be better understood.
• The role of inequality of educational opportunity at time of schooling (IEO) for later-life cognition is not yet clear.
• In a sample from 16 European countries and Israel, higher IEO predicted lower later-life cognitive functioning.
• Particularly women with lower education of the cohorts under investigation seem to have been vulnerable to higher IEO.

## Introduction

Unimpaired cognitive functioning is an important determinant of healthy aging and is shaped from very early ages on (Richards & Hatch, 2011). Schooling is among the most well-established factors to influence older-age cognitive outcomes, such as cognitive decline (Zahodne et al., 2011). In fact, recent research suggests that education before age 20 is the main contributing factor to increasing cognitive skills (Kremen et al., 2019). From a contextual perspective, schooling systems have been identified as important determinants of cognitive skills in childhood and adolescence, the role of which can be conceptualized with inequality of educational opportunity to explain student cognitive outcomes (Burger, 2016; Gamoran & Mare, 1989). Inequality of educational opportunity (IEO) describes the extent to which schooling opportunities depend on social origins, i.e. parental educational or socioeconomic background, rather than student cognitive skills. IEO is measured by the country- and cohort-specific correlation between length of schooling of members of a birth cohort and that of their parents (Rotman et al., 2016). To this date, little is known about how far-reaching into adulthood and older age the effects of IEO on cognitive functioning are, particularly if levels of IEO at time of schooling systematically influence cognitive functioning in later mid- and older adulthood.

This study is building upon well-established evidence that contextual determinants, that is, characteristics specific to country and historical period, influence older-age cognitive functioning: Earlier studies have identified macro-level determinants of cognitive functioning such as compulsory schooling (Glymour et al., 2008; Schneeweis et al., 2014), distribution in educational attainment (Olivera et al., 2018), and exposure to economic recessions that come with limited work opportunities (Leist et al., 2014). Testing a new potential macro-level determinant, this study aims to assess the contribution of IEO at time of schooling to cognitive functioning at later mid-adulthood and early older age, and rate of cognitive decline with age.

IEO is hypothesized to systematically alter the degree of cognitive stimulation during schooling. Schooling has been well established to contribute to cognitive development and cognitive functioning across the life span (Lövdén et al., 2020). Prolonged exposure to or engagement in cognitively stimulating activities such as education, occupational level, or occupational complexity have been shown to be associated with higher levels of cognitive functioning at older ages (Andel et al., 2006; Ritchie & Tucker-Drob, 2018; Singh-Manoux et al., 2011).

Further, IEO is specific to country and historical time (cohort) and thus an exposure with temporal precedence and exogeneous to individual-level cognitive performance. Therefore, reverse causation can be ruled out, and any associations between levels of IEO and cognitive outcomes can be suggested to be produced by IEO-associated differences in schooling opportunities and thus the cognitive development during schooling.

Specifically, we hypothesize that higher levels of IEO hinder at the time of schooling cognitive development of pupils, and lead to both lower levels of cognitive functioning and steeper decline in cognitive functioning with age, both of which are relevant outcomes: Lower cognitive skills earlier in life are associated with diminished opportunities later in life (Heckman et al., 2006), and mean a lower starting point at the onset of cognitive aging in mid-adulthood (Singh-Manoux et al., 2012). The rate of cognitive decline with age is relevant as steeper decline would lead to reaching a threshold of cognitive impairment at an earlier age (Lövdén et al., 2020). The chosen methodological approach of multilevel (mixed-effects) models enables us to pursue the impact of levels of IEO on both cognitive functioning and decline simultaneously.

In the following, we will argue that schooling systems, and more specifically, the level of IEO, systematically impacts older-age cognitive functioning.

On the individual level, the most important contributing factors to cognitive development are indeed the length and quality of education (Foverskov et al., 2018; Glymour et al, 2008, 2012; Langa et al., 2017). There is compelling evidence that schooling, conceptualized as period of extended cognitive stimulation across diverse activities aiming at increasing analytical, language, mathematic, and other skills, increases cognitive skills in a dose-response relationship (Lager et al., 2017; Ritchie & Tucker-Drob, 2018).

On the contextual level, contexts with higher IEO may provide children from more advantaged socioeconomic backgrounds better educational resources than children from disadvantaged backgrounds (Burke, 1999; Coleman, 1968). In contexts with lower IEO, education systems should be more equitable, and children independent of socioeconomic backgrounds should receive more education that matches their skills. Indeed, stronger skill-mismatching is present in higher-IEO countries (Esping-Andersen & Cimentada, 2018).

As such, early-life cognitive abilities determine possibilities for educational attainment to a considerable extent (Deary & Johnson, 2010), underlining the importance of adequate, that is, skill-matching, education during a time where neuroplasticity is higher. We assume that there is an optimal match between a child's innate cognitive skills and the length and complexity of schooling the child may receive to reach optimum levels of cognitive performance.

Adding a sex/gender dimension to the study will incorporate relevant differences in the observed concepts and associations under investigation. A sex/gender dimension is relevant for several reasons: First, earlier gender gaps in educational attainment have, as long-lasting trends, reduced over the last decades due to modernization of society, which, in some cases, have even reversed (Bar-Haim et al., 2018; Treiman et al., 2003). The cohorts under investigation in this study have participated in this trend.

Further, IEO has been shown to have differential effects on men and women in other studies (Bar-Haim et al., 2019; Karmaeva et al., 2020; Manzoni, 2020), and we suspected sex/gender differential effects on cognitive functioning as well.

Lastly, cognitive functioning at older ages is shaped by societal context such as gender-role attitudes. Recent research has shown that the sex/gender differential, that is, the extent to which there is a female advantage in cognitive functioning, varies according to the status of women in society, with more egalitarian views being associated with higher female advantage in memory (Bonsang et al., 2017).

## Research questions

Objectives of this study are the following: (1) to quantify the associations of IEO at time of schooling and older-age cognitive functioning and rate of cognitive decline with age, and (2) to test if these associations differ between men and women in general, and between men and women with different educational attainment.

We assume that in contexts with higher IEO, students from socioeconomically less advantaged parental backgrounds will have fewer opportunities to attain appropriate levels of education that they could attain based on their cognitive skills. Additionally, children from more advantaged backgrounds may be exposed to less pressure to pursue the cognitively stimulating activities offered and graded during schooling as these would be less relevant to reach appropriate educational attainment compared to more meritocratic societies. Conversely, in more meritocratic, lower-IEO contexts, education in primary and lower secondary school may on average be more demanding to prepare all children adequately to pursue higher educational attainment.

We thus assume that respondents schooled in higher-IEO contexts linked will show lower cognitive functioning and steeper rate of cognitive decline with age in later mid- and older adulthood. Additionally, we will explore sex/gender differences in these associations.

## Methods

### Data

Individual-level data came from the Survey of Health, Ageing and Retirement in Europe (SHARE), a longitudinal survey of representative samples of the population aged 50 and older that started in 2004, with biennial assessments in initially 14 European countries, with the number of participating countries growing over time (Börsch-Supan, 2019; Börsch-Supan & Gruber, 2019). The latest data used in these analyses were collected in Wave 7 in 2017. SHARE is an interdisciplinary survey that comprises comparable information on health, employment, social conditions, and life histories. The survey and the available data are described in detail elsewhere (Börsch-Supan et al., 2013; Börsch-Supan & Jürges, 2005; Gruber, 2019). Wave 3 (SHARELIFE, 2008/9) was dedicated to assessing life histories, and did not assess cognitive functioning. Wave 5 included a mini-childhood module. Wave 7 similarly assessed life histories (SHARELIFE) of all respondents who did not participate in Wave 3; those who did received a classic SHARE survey. Ethical standards, study design, and data collection of the SHARE survey were approved by the internal review board (IRB) at the University of Mannheim, Germany (Börsch-Supan & Jürges, 2005). Ethical standards of the CRISP Cognitive Aging research project that this study is part of were approved by the Ethics Review Committee of the European Research Council in November 2018.

The full sample selection flow is illustrated in Appendix Fig. 1. A total of *N* = 72,888 respondents participated in two regular SHARE (non-SHARELIFE) waves and provided at least two cognitive measurements. Of these, *N* = 49,933 belonged to countries and cohorts for which information on levels of IEO at time of schooling was available, and *N* = 46,448 reported information on all relevant covariates. Up to six cognitive assessments over 13.5 years were available.

Data on inequality of educational opportunity came from the World Bank Global Database on Intergenerational Mobility (GDIM) (GDIM, 2018) and were available for the cohorts born 1940–49 (cohort 1), 1950–59 (cohort 2), and 1960–63 (cohort 3) in the participating SHARE countries of Austria, Germany, Switzerland, Belgium, Netherlands, Denmark, Sweden, Czech Republic, Poland, Estonia, Portugal, Spain, France, Italy, Greece, and Israel. Data on Luxembourg were not represented in the GDIM. We excluded individuals schooled in a country different from the country of residence after the age of 10 years (*N* = 1386 individuals).

The youngest cohort born 1960+ was not represented in the SHARE data of Greece (0 cases) and insufficiently in Poland (2 cases), leading to a total of 49 country-cohorts. After excluding two observations of one female respondent with a standardized cognitive score >2*SD* above the mean of the reference country-cohort, data of *N* = 49,933 respondents born between 1940 and 1963 entering SHARE at the latest in 2013 at age 50) provided at least two measurements of cognitive functioning, totaling 173,269 observations.

Country-cohort sizes ranged between 45 (cohort 3 of Netherlands) and 48 (cohort 3 of Portugal) to 1973 (cohort 1 of Sweden) and 2195 (cohort 1 of Czech Republic), see Appendix Fig. 2 for a visualization of the distribution of the range of IEO values and sample sizes.

Of this sample, a total of *N* = 46,972 had information on individual education, partner situation, and current job situation available, and *N* = 46,448 (25,544 women and 20,904 men) additionally provided information on health, chronic conditions, and depressive symptoms, with on average *M* = 2.42 (*SD* = 1.27) assessments and *k* = 161,560 observations in total.

Among the respondents to the regular SHARE interviews who also participated in one of the SHARELIFE Waves 3 or 7, a subsample of N = 29,442 provided information on childhood health, cultural capital, and school performance in mathematics and language, respectively, relative to their peers. Any small fluctuations in the sample sizes come from missing values on one of the three cognitive indicators.

### Individual-level measures

**Outcome**. Cognitive function was assessed in up to six assessments through measures of executive function and memory (Dewey & Prince, 2005). Executive function (verbal fluency) was assessed by asking respondents to name as many animals as possible in 1 min. Memory was assessed by listening to a 10-word list read aloud by the interviewer, after which respondents were asked to repeat the words immediately (immediate recall) and after a standardized period of time (delayed recall). The three measures have been shown to be susceptible to aging, and verbal fluency additionally is sensitive to changes in health (Mazzonna & Peracchi, 2013).

In Waves 1 and 2, the same word list was used for both waves. For Wave 4 and the subsequent waves, three new, different lists were administered alternatingly to minimize practice effects. Main models are reported with the z standardized single outcomes. Cognitive scores of all waves were standardized with Wave 1 mean and standard deviation, separately by sex/gender and cohort.

**Education**. Education was derived from ISCED-97 categories, with categories up to lower secondary (ISCED 0-2), upper secondary (3), and post-secondary or tertiary education (4–6).

**Current self-rated health**. Respondents rated their health between 1 “excellent” and 5 “poor”. We built an indicator to assess the reporting of poor health.

**Depressive symptoms** were assessed with the EURO-D scale (range 0–12). Example items are “in the last month, have you been sad or depressed?” or “Have you had trouble sleeping recently?“.

**Chronic conditions** were asked in the “has a doctor told you that you had” format, and assessed if participants had ever been diagnosed with a heart attack including myocardial infarction or coronary thrombosis, high blood pressure, high blood cholesterol, stroke or cerebral vascular disease, diabetes or high blood sugar, chronic lung disease, cancer, stomach or duodenal ulcer, Parkinson's disease, cataracts, or hip fracture. Responses were summed (range 0–9 out of maximum 14 conditions).

**Current job situation** was assessed at the entry wave and had five categories: 1 “retired”, 2 “employed or self-employed”, 3 “unemployed”, 4 “permanently sick or disabled”, 5 “homemaker”. The category “other” was set to missing due to very low overall prevalence of this job status.

### Childhood covariates

Harmonized childhood information was assessed in waves 3, 5, and 7.

**Number of books at age 10 in parental household** was assessed as proxy for parental cultural capital (De Graaf et al., 2000; Esping-Andersen, 2004). This indicator also partially assesses parental socioeconomic background (Brunello et al., 2017; Havari & Mazzonna, 2015) and a cognitively active lifestyle at early ages (Wilson et al., 2005). Categories ranged from 1 “none or very few (0–10)" books to 5 “enough to fill two or more bookcases (more than 200)" books. Less advantaged parental socioeconomic conditions have recently been shown to predict higher cognitive decline particularly in women (Wolfova et al., 2021), although evidence is mixed (Aartsen et al., 2019).

**School performance** Respondents rated their relative school performance in mathematics and language, respectively, relative to peers, at age 10, on a scale ranging from 1 “much better” to 5 “much worse”. School performance has been shown to be predictive of dementia in a U.S. sample (Mehta et al., 2009).

**Childhood health** Respondents rated their health in childhood at age 10 between 1 “excellent” and 5 “poor”. The category “health varied a great deal” (*n* = 53, 0.1%) was set to missing.

### Macro-level determinants and confounders

**Country-level inequality of educational opportunity** was provided by the World Bank Global Database on Intergenerational Mobility (GDIM, 2018; Narayan et al., 2018), specifically Pearson's correlation coefficient between parents' and children's years of education for 10-year cohorts born after 1940 (variable COR). The higher the coefficient, the more intergenerational persistence, that is, higher IEO (Bernardi & Ballarino, 2016; Rotman et al., 2016).

**Healthy life expectancy at older age (HLE)**. From the WHO Global Health Data Repository (WHO Global Health Data Repository, 2019), healthy life expectancy at age 60 was used as an indicator for the average health status of older people in the different countries in 2005. The indicator is computed as average expectation of life years in “full health” at age 60 across different health states, adjusted for severity distribution, and sensitive to change over time and differences between countries.

**Gross domestic product per capita (GDP).** The wealth or standard of living of each country was evaluated by the purchasing power adjusted value in international dollars available from the World Development Indicators for 2004 (World Bank, 2019).

**Human Development Index (HDI)**. The HDI is composed to indicate a long and healthy life, knowledge, and a decent standard of living. It is calculated as a composite of life expectancy at birth, both expected and actual mean years of schooling, and gross national income per capita (PPP $). Data were taken from UN Human Development Reports of 2005. The HDI is as such the strongest indicator of the general economic, health, and educational capital of a country.

### Strategy of data analysis

Participants with at least two and up to six cognitive measurements were included to be able to capture cognitive change and to better approximate “true” cognitive performance (Singh‐Manoux et al., 2011).

In a first step, the bivariate association between level of IEO and the difference between female and male aggregate country-cohort starting levels of cognitive functioning (average of the three z-standardized cognitive measures) were plotted to inspect the distribution for possible outliers that would drive a potential association.

We used multilevel (also called mixed-effects or hierarchical linear) modeling to investigate the impact of contextual influences on cognitive levels and decline (Glymour et al., 2012; Lavrencic et al., 2018). In doing this, we are able to distinguish between unsystematic variation in cognitive functioning (captured by random intercepts on the individual and country level, and able to adjust for language and other unobserved country-level determinants) and systematic variation due to individual education or contextual IEO (captured by the coefficients of the so-called fixed effects on different levels). Within-person change in cognitive functioning, that is, the individual-level slope of change, is estimated by the coefficient of age at measurement, which indicates distance between measurements.

The effect of the exposure IEO on cognitive functioning was assessed as main effect, the effect of IEO on cognitive decline (rate of change) was assessed by modeling an interaction term of the exposure IEO times age at measurement; the coefficient of IEO*age estimates the effect of IEO on within-person change between measurements (Glymour et al., 2012).

Multilevel (mixed-effects) models were run with level 1 being observations nested within individuals, level 2 being individuals nested within country-cohorts, and level 3 being country-cohorts.

We specified random intercepts on the individual level, accounting for varying individual starting values in cognitive measures, as well as on the country-cohort level. In a cross-country investigation of cognitive performance, the random country-(cohort) level intercept captures language differences in the measurement of the cognitive outcomes, as well as other unmeasured macro-level determinants, i.e., heterogeneity by country and the historical period (Leist et al., 2013). Specifying a random effect for age did not change the result patterns in the simpler models, but led to failures of convergence in some of the more complex models, so it was removed from the analyses presented here.

In line with the assumptions of sex/gender-specific associations of level of IEO and cognitive functioning, we stratified models by sex/gender, and tested the three-way interaction of level of IEO with sex/gender and education on the three indicators of cognitive functioning.

All variables were centered at their mean, except age which was centered at age 50 and divided by 10; the resulting coefficient indicated average cognitive change per decade after age 50. A centered age-squared term was entered to account for nonlinear trajectories and improved model fit. An indicator for first assessment was included to adjust for cognitive practice effects (Vivot et al., 2016; Weuve et al., 2015).

Childhood and current health, number of books, school performance, education, job situation, and cohabitation status were entered as categorical variables into the models. Age, age-squared, number of depressive symptoms, and chronic conditions, respectively, and the contextual variables IEO, HDI, GDP, and HLE were treated as continuous variables.

We followed a step-wise model building process, first testing random intercepts and slopes at the two upper levels, then subsequently including the different covariates.

Model 1 included main effects of levels of IEO on cognitive levels and rate of cognitive decline (IEO*age), cohabitation status (married or living together vs. separated, widowed, divorced), centered age in decades, centered age-squared, education in three categories, current job situation, self-rated health in five categories, number of depressive symptoms, and number of chronic diseases.

Model 2 was run on the subsample with available childhood information (SHARELIFE interviews in Waves 3 and 7), and included Model 1 covariates plus childhood health, number of books at age 10, school performance in mathematics and language, respectively, relative to peers. Model 3 included individual controls of Model 1 and, one at a time, competing contextual determinants (GDP, HLE, HDI). We report the results of the models including HDI, as this is the most relevant covariate indicating a joint assessment of average life expectancy, standard of living, and education of the countries.

For interaction analyses, sex/gender was entered as an indicator coded 1 for “male” and 0 for “female".

In a first step, the two-way interactions between level of IEO and sex/gender and IEO and education (three levels) were tested. We report the results of the full model, additionally specifying a three-way interaction of IEO with education and sex/gender. Two-way interaction coefficients were similar in the full model.

Analyses were carried out with R, particularly with the lmer package (Bates et al., 2014; Kuznetsova et al., 2017; Lüdecke, 2019; Nash, 2014; Wickham, 2016).

## Results

### Descriptive findings

The main findings are based on subsequent model building with a total of initially *N* = 46,448 respondents with two or more cognitive assessments and information on covariates. We ran models separately for 25,544 women and 20,904 men.

Respondents were aged 50–76 years at first measurement. Mean age of respondents in each country-cohort ranged between 50.7 years (Estonia 1960–63) and 67.1 years (Slovenia 1940–49) at first assessment.

About one-third had up to lower secondary education (38.2% of women, 33.3% of men) or upper secondary education (34.5% of women, 36.6% of men), and about one-fourth had post-secondary or tertiary education (27.3% of women, 30.1% of men, Table 1). A total of 16,106 men and 20,426 women provided three cognitive assessments, 9069 men and 12,275 women four assessments, 3183 men and 4140 women five assessments, and 1511 men and 1954 women six assessments. Median follow-up time was 6.09 years (IQR 4.10, 10.4).

**Table 1:** Demographic characteristics of the sample.

	Women (*n* = 25,544)	Men (*n* = 20,904)
	*M* (*SD*)/*N* (%)	*M* (*SD*)/*N* (%)
Age (50–76 years)	59.3 (6.00)	59.6 (5.97)
Education		
Up to lower secondary (ISCED 0–2)	9751 (38.2%)	6952 (33.3%)
Upper secondary (ISCED 3)	8813 (34.5%)	7657 (36.6%)
Post-secondary and tertiary (ISCED 4–6)	6980 (27.3%)	6295 (30.1%)
Self-rated health		
Fair or better	23,671 (92.7%)	19,472 (93.1%)
Poor	1873 (7.3%)	1432 (6.9%)
Number of chronic diseases (0–9)	0.933 (1.10)	0.993 (1.12)
Number of depressive symptoms (0–12)	2.64 (2.27)	1.81 (1.90)
Childhood variables retrospective at age 10		
Number of books in household		
None or very few (0–10 books)	5398 (21.1%)	4586 (21.9%)
Enough to fill one shelf (11–25 books)	4975 (19.5%)	4082 (19.5%)
Enough to fill one bookcase (26–100 books)	6068 (23.8%)	4882 (23.4%)
Enough to fill two bookcases (101–200 books)	2301 (9.0%)	1718 (8.2%)
Enough to fill two or more bookcases (more than 200 books)	2570 (10.1%)	1898 (9.1%)
Missing	4232 (16.6%)	3738 (17.9%)
Mathematical skills relative to peers		
Much better	1411 (5.5%)	1426 (6.8%)
Better	4118 (16.1%)	3960 (18.9%)
About the same	11,964 (46.8%)	9200 (44.0%)
Worse	3126 (12.2%)	2171 (10.4%)
Much worse	604 (2.4%)	394 (1.9%)
Missing	4321 (16.9%)	3753 (18.0%)
Language skills relative to peers		
Much better	1828 (7.2%)	926 (4.4%)
Better	5491 (21.5%)	3137 (15.0%)
About the same	11,556 (45.2%)	9750 (46.6%)
Worse	2026 (7.9%)	2938 (14.1%)
Much worse	291 (1.1%)	374 (1.8%)
Missing	4352 (17.0%)	3779 (18.1%)

*Note*. M, mean, SD, standard deviation, N, sample size.

Testing for selective attrition, a total of 3.4% of respondents in the final sample were known to have died over the course of the study (*n* = 1,610, 3.4%). Respondents who had died at one of the follow-ups were more likely to be older (OR 1.05, CI 1.04, 1.06), more likely to be male (female: OR 0.46, CI 0.42, 0.51), and less likely to be higher educated (ISCED 3: OR 0.77, CI 0.69, 0.87; ISCED 4–6: OR 0.46, CI 0.40, 0.53).

Performance on the cognitive measures declined with age with an increasingly negative slope.

IEO, that is, the correlation between parental and offspring's years of education on the population level, ranged from *r* = 0.298 and *r* = 0.312 in the 1960–63 cohorts of the Netherlands and Denmark, up to *r* = 0.641 and *r* = 0.652 in the 1940–49 cohorts of Portugal and Italy.

### Impact of level of IEO on level of cognitive functioning

In most models, higher levels of IEO were associated with lower levels in the cognitive measures in men and women (Table 2).

**Table 2:** Associations of level of IEO on level of and rate of change in three cognitive measures in stratified multilevel (mixed-effects) models.

	Women			Men			
	Immediate recall	Delayed recall	Verbal fluency	Immediate recall		Delayed recall	Verbal fluency
	Coeff. (CI)	Coeff. (CI)	Coeff. (CI)	Coeff. (CI)		Coeff. (CI)	Coeff. (CI)
(Intercept)	−0.27 *** (−0.34 to −0.19)	−0.20 *** (−0.28 to −0.12)	−0.29 *** (−0.39 to −0.19)	−0.30 *** (−0.37 to −0.24)		−0.26 *** (−0.33 to −0.19)	−0.25 *** (−0.35 to −0.16)
Age centered at 50 in decades	0.15 *** (0.11–0.19)	0.17 *** (0.12–0.21)	0.09 *** (0.05–0.13)	0.18 *** (0.13–0.23)		0.20 *** (0.15–0.25)	0.03 (-0.02 – 0.08)
Age squared	−0.10 *** (−0.11 to −0.08)	−0.10 *** (−0.12 to −0.09)	−0.07 *** (−0.08 to −0.05)	−0.11 *** (−0.13 to −0.09)		−0.11 *** (−0.12 to −0.09)	−0.05 *** (−0.07 to −0.04)
Indicator of first testing	−0.08 *** (−0.09 to −0.06)	−0.12 *** (−0.13 to −0.11)	−0.05 *** (−0.06 to −0.03)	−0.06 *** (−0.08 to −0.04)		−0.10 *** (−0.12 to −0.09)	−0.05 *** (−0.06 to −0.04)
Education (reference: ISCED 0–2)							
ISCED 3	0.36 *** (0.34–0.38)	0.34 *** (0.32–0.36)	0.32 *** (0.30–0.34)	0.30 *** (0.27–0.32)		0.26 *** (0.24–0.29)	0.25 *** (0.22–0.28)
ISCED 4–6	0.60 *** (0.58–0.62)	0.59 *** (0.57–0.62)	0.62 *** (0.59–0.64)	0.56 *** (0.54–0.59)		0.54 *** (0.52–0.57)	0.50 *** (0.47–0.52)
Job situation (reference: Retired)							
Employed or self-employed	0.10 *** (0.08–0.12)	0.11 *** (0.08–0.13)	0.10 *** (0.07–0.12)	0.09 *** (0.06–0.11)		0.11 *** (0.08–0.14)	0.08 *** (0.05–0.11)
Unemployed	−0.01 (-0.05 – 0.04)	−0.00 (-0.05 – 0.04)	−0.06 * (−0.10 to −0.01)	−0.05 * (−0.09 to −0.00)		−0.03 (-0.08 – 0.02)	−0.08 *** (−0.13 to −0.04)
Permanently sick or disabled	−0.11 *** (−0.16 to −0.07)	−0.10 *** (−0.14 to −0.05)	−0.12 *** (−0.17 to −0.07)	−0.15 *** (−0.20 to −0.10)		−0.11 *** (−0.16 to −0.06)	−0.18 *** (−0.23 to −0.13)
Homemaker	−0.04 * (−0.06 to −0.01)	−0.03 (-0.06 – 0.00)	−0.05 *** (−0.08 to −0.02)	0.02 (-0.13 – 0.17)		0.06 (-0.10 – 0.23)	−0.03 (-0.19 – 0.14)
Cohabitation status (reference: Living alone)							
Cohabiting with partner	0.04 *** (0.02–0.06)	0.00 (-0.02 – 0.02)	0.05 *** (0.03–0.07)	0.08 *** (0.05–0.10)		0.06 *** (0.03–0.08)	0.09 *** (0.06–0.11)
Self-rated health (reference: Fair or better)							
Poor	−0.17 *** (−0.20 to −0.13)	−0.16 *** (−0.19 to −0.12)	−0.16 *** (−0.19 to −0.12)	−0.19 *** (−0.23 to −0.14)		−0.17 *** (−0.21 to −0.13)	−0.16 *** (−0.20 to −0.11)
Number of chronic diseases (0–9)	−0.02 *** (−0.03 to −0.02)	−0.03 *** (−0.03 to −0.02)	−0.03 *** (−0.04 to −0.02)	−0.00 (-0.01 – 0.01)		−0.01 (-0.02 – 0.00)	0.00 (-0.01 – 0.01)
Number of depressive symptoms (0–12)	−0.02 *** (−0.03 to −0.02)	−0.02 *** (−0.03 to −0.02)	−0.02 *** (−0.03 to −0.02)	−0.03 *** (−0.04 to −0.03)		−0.03 *** (−0.04 to −0.03)	−0.03 *** (−0.04 to −0.03)
IEO	−1.23 ** (−1.97 to −0.48)	−0.97 * (−1.78 to −0.16)	−1.77 ** (−2.84 to −0.70)	−0.94 ** (−1.50 to −0.38)		−0.60 (-1.20 to −0.00)	−1.79 *** (−2.74 to −0.84)
IEO*age	0.17 * (0.02–0.32)	−0.17 * (−0.32 to −0.02)	−0.39 *** (−0.53 to −0.24)	0.48 *** (0.32–0.65)		0.01 (-0.16 – 0.18)	−0.16 (-0.32 – 0.01)
**Random Effects**							
σ^2^	0.55	0.55	0.41	0.58		0.57	0.45
τ_00 individual-level_	0.26	0.32	0.32	0.27		0.32	0.35
τ_00 country-cohort_	0.05	0.06	0.11	0.02		0.03	0.08
ICC	0.36	0.41	0.51	0.33		0.38	0.49
N	25,544	25,544	25,538	20,904		20,903	20,900
Number of country-cohorts	49	49	49	49		49	49
Observations	89,713	89,748	77,284	71,455		71,525	61,807
Marginal R^2^/Conditional R^2^	0.130/0.440	0.124/0.483	0.173/0.596	0.096/0.398		0.092/0.439	0.120/0.553

*Note*. *p < 0.05. **p < 0.01. ***p < 0.001. Std., standardized, CI, confidence interval, ISCED, International Standard Classification of Education, No., number, IEO, Inequality of educational opportunity, σ^2^, mean random effect variance (overall), τ_00_, random effect (between-units) variances on individual level and country-cohort level, ICC, Intra-class correlation, N, number of respondents, Marginal R^2^, variance explained by fixed effects, Conditional R^2^, variance explained by fixed and random effects.

Higher level of IEO was associated with lower levels of immediate recall and verbal fluency in men (immediate recall: Coeff. −0.94, CI -1.50, −0.38; verbal fluency: Coeff. −1.79, CI -2.74, −0.84; Table 2), but no associations with delayed recall were present (*n.s.*)

In women, higher IEO was associated with lower levels of performance in all three cognitive measures (immediate recall: Coeff. −1.23, CI -1.97, −0.48; delayed recall: Coeff. −0.97, CI -1.78, −0.16; verbal fluency: Coeff: 0.39, CI -0.53, −0.24; Table 2).

### Impact of level of IEO on rate of cognitive decline

Testing the association of IEO*age and measures of cognitive functioning to investigate the effect of level of IEO on cognitive decline, generally, patterns suggest that IEO did not affect cognitive decline in women and men equally (Table 2). Over the follow-up of up to 13.5 years, men schooled in higher-IEO contexts showed slower decline in immediate recall (Coeff. 0.48, CI 0.32, 0.65) and no effects of IEO on rate of decline in delayed recall and verbal fluency, suggesting overall an absence of effects of IEO on acceleration of cognitive decline in men.

In contrast, women schooled in higher-IEO contexts showed more decline in delayed recall and verbal fluency compared to women schooled in lower-IEO contexts (delayed recall: Coeff. −0.17, CI -0.32, −0.02; verbal fluency: Coeff: 0.39, CI -0.53, −0.24). Associations of level of IEO and decline in immediate recall in women were not significant.

### Sex/gender differences in the impact of level of IEO on cognitive functioning

In a next step, we tested if men and women of different educational levels would show different associations with the three indicators of cognitive functioning, depending on how strong IEO was at the time of schooling. In the pooled sample testing the two- and three-way interactions of level of IEO, education, and sex/gender, the main effect of level of IEO on the cognitive level was only significant in verbal fluency (Coeff. −1.73, CI -2.76, −0.71; see the full set of coefficients in Appendix Table 4). Regarding the two-way interactions, women showed lower cognitive scores in all three measures if schooled in higher-IEO contexts. Overall, the strength of the coefficients of educational levels suggested lower differences between verbal fluency performance of respondents of different educational levels if schooling took place in higher-IEO contexts (IEO*ISCED 3: Coeff. −0.38, CI -0.66, −0.10; IEO*ISCED 4–6: Coeff. −0.35, CI -0.64, −0.05). The three-way interactions were significant, showing that women of higher educational levels who were schooled in higher-IEO contexts performed higher on the three cognitive tests compared to women of lower educational level on all three outcomes (e.g., immediate recall: IEO*female*ISCED 3: Coeff. 0.80, CI 0.46, 1.14; IEO*female*ISCED 4–6: Coeff. 0.83, CI 0.47, 1.19; Appendix Table 4).

### Robustness checks

Additional analyses in the subsample with information on childhood health, socioeconomic status, and school performance replicated the main effect of level of IEO on measures of cognitive functioning presented in Table 2 in most cases (Appendix Table 1), as did the additional analyses in the subsample with information on occupational level of current or last job if retired or sick/disabled, respectively (Appendix Table 2). Regarding effects of level of IEO on rate of change in the measures of cognitive functioning, results for men were consistent when additionally controlling for childhood or occupational information (Table 1, Table 2). However, the effect of level of IEO on decline in delayed recall and verbal fluency in women disappeared after adjusting for childhood measures or occupation, suggesting that only women schooled in higher-IEO contexts with less advantaged parental socio-economic background and lower occupational status had steeper cognitive decline (Table 1, Table 2). Analyses controlling additionally for HLE, GDP or HDI (one at a time) largely confirmed the main analyses presented in Table 2 (Appendix Table 3) except for the effect of level of IEO on delayed recall in women, which was not significant anymore after entering HDI.

Inspecting the lmer correlation matrix, the correlations between the coefficients of IEO and IEO*age were negligible in all models, suggesting that the associations of IEO*age and the cognitive performance measures were not driving the findings. Additionally controlling for the number of children did not change the result patterns. As a quadratic age effect was modelled, additional models tested a separate interaction term of IEO*age squared. In the models that included this interaction term, the coefficients of age, age squared, IEO, and IEO*age did not change in direction or significance. In four of the six models (three cognitive measures in men and women), the coefficient of the new term (IEO*age squared) was not significant; in two models it was small and positive, suggesting a slightly softening negative effect of level of IEO with age. As the coefficients of the other effects did not change, for clarity of interpretation this term was not included in the final models presented in Table 2.

## Discussion

### Explanation of findings

In 49 cohorts of 17 developed countries, higher inequality of educational opportunities at the time of schooling was associated with lower immediate recall, delayed recall, and verbal fluency scores in older age, most consistently in women. Controlling for health and work variables, interaction analyses confirmed worse cognitive functioning of women who received lower levels of education in higher-IEO contexts. Most results held after controlling for additional contextual determinants indicating country-level health and economic development, and in the subsample with information on childhood health, socioeconomic status, and school performance. The results provide support for the hypothesis that higher-IEO contexts may hinder some children to receive skills-matching education, and are in line with recent findings of significant skill-mismatching in high-IEO countries (Esping-Andersen & Cimentada, 2018). This could have profound long-lasting consequences for later-life cognitive functioning, particularly for lower-educated women of the cohorts and countries under investigation.

Suggested mechanisms to explain these findings are that in less meritocratic, higher-IEO contexts, there will be different practices in educational systems. These practices comprise tracking, that is, grouping pupils according to ability into different subjects or curricula within a school; sorting, that is, assign grades, and labeling through category labels to signal pupils’ ability (Domina et al., 2017; Stiglitz, 1975). Schooling systems may, through educational practices such as tracking, create higher IEO (Lucas, 2001; Skopek et al., 2019) by labeling students more according to *origins*, particularly parental education, compared to more meritocratic systems that focus instead on *cognitive skills* independent of origins. Put differently, since students assigned to higher tracks benefit more in terms of academic skills, tracking may reinforce existing inequalities between students coming from different socioeconomic backgrounds (Gamoran & Mare, 1989). In line with these findings, in another study, highly tracked educational systems (representing more unequal schooling systems) were associated with the steepness of the achievement gradient between children from lower- and higher-educated parental backgrounds (Burger, 2016). While these mechanisms of tracking, sorting, and labeling could not be assessed directly, our findings lend support to the hypothesis that stronger skill-mismatching may have occurred in higher-IEO contexts, with associated long-term disadvantages for cognitive development.

Women seem to have been more disadvantaged in more unequal countries in terms of cognitive development, which is reflected in comparatively lower cognitive functioning (for cohorts born between 1940 and 1963), in line with earlier findings (Bonsang et al., 2017).

Generally, women from all socioeconomic backgrounds are doing better than men at school, and women's rates in higher education have even surpassed those of men in the last decades (DiPrete & Buchmann, 2013). In higher-IEO contexts, it is possible that women's comparative advantage cannot be realized due to the nonmeritocratic nature of these systems. The interaction models presented here suggest that higher-IEO contexts may prevent women, particularly from lower educated backgrounds who are at increased risk to not receive skill-matching education, to reach their full cognitive potential. The models incorporating childhood information showed that women schooled in higher-IEO contexts and who had less advantaged parental socioeconomic backgrounds had steeper cognitive decline, confirming and extending recent findings (Wolfova et al., 2021). Further, several studies link changes in educational system to changes in gender egalitarianism (Shu, 2004; Thijs et al., 2019). Hence, one could speculate about a link of higher IEO with less egalitarian gender-role attitudes that may be either reflected in teacher practices to focus more on boys' cognitive development than that of girls, or even hurt women's cognitive development during schooling. However, to our knowledge a systematic analysis on this topic has not been conducted.

In most studies that investigated the impact of social and socioeconomic determinants on rate of cognitive decline, only small associations were observed, often only with subdomains for cognitive functioning, which cannot be fully explained to date (Glymour et al., 2012; Lavrencic et al., 2018; Seblova et al., 2020). Earlier studies found sex/gender differences in cognitive decline with age to be small or nonexistent (Aartsen et al., 2004; Glymour et al., 2012). If anything, women showed greater resilience to cognitive aging than men (McCarrey et al., 2016). However, effects of early-life socioeconomic disadvantage on cognitive performance have been shown to be greater for women than men (Wolfova et al., 2021). In this study, we observed that the association of level of IEO with rate of cognitive decline differed between the sexes: Associations of level of IEO with rate of cognitive decline were largely absent in men, whereas women showed steeper decline in two measures of cognitive performance, delayed recall and verbal fluency. This suggests that cognitive development of women schooled in higher-IEO contexts was lower than that of women schooled in lower-IEO contexts. Particularly women with less advantaged socioeconomic background may have greater benefits from education, in line with theory of resource substitution (Ross & Mirowsky, 2006), which they may not have been able to realize in higher-IEO contexts during schooling.

Regarding rate of decline across the cognitive measures, women showed steeper decline in delayed recall as an indicator of memory, and verbal fluency that assesses executive functioning. Memory and executive functioning may have higher potential to be increased through cognitively stimulating activities than attentive measures. Memory in particular is influenced by length of schooling (Glymour et al., 2008). The animal naming test includes a strong language component and benefits from the use of strategies, both skills that are increased during schooling (Da Silva et al., 2004; Whiteside et al., 2016). Attentive measures such as immediate recall may possibly be less susceptible to the benefits of schooling. As pointed out above, we still lack solid knowledge on the drivers of decline in cognitive performance with aging (Lavrencic et al., 2018; Lövdén et al., 2020; Marinescu et al., 2020).

Although we found increasingly negative decline in all measures of cognitive functioning with age, in line with earlier findings (Salthouse, 2010), an additional explanation for the absence of effects of level of IEO on cognitive decline in men could be that our study sample was in quite young old age, with largely absent clinically relevant cognitive decline. An older sample may possibly convey effects of level of IEO on cognitive decline in men as well.

We tested the contribution of occupational level of current and last job if currently retired or unemployed, respectively, but associations with cognitive performance did not change. This is in line with the greater importance of education compared to occupational pathways after age 20 (i.e., after most of schooling has been completed) for cognitive development (Kremen et al., 2019).

The exact mechanisms through which IEO acts cannot be fully established due to a lack of information on the educational practices to which respondents have been exposed during schooling. However, we confirm here the long lasting effects of inequality in childhood, and their consequences across the life course, reflecting durable ‘cumulative chains of adversities’ experienced by the most vulnerable populations (Spini et al., 2017). Earlier studies suggested that higher IEO is more prominent in countries with more strongly differentiated, that is, school-type tracked, educational systems, while lower IEO is more prominent in countries with more standardized educational systems (Van de Werfhorst & Mijs, 2010). Our study suggests that, through these practices, IEO systematically impacts potential for cognitive development on a cohort level.

From a policy perspective, eliminating IEO is hardly possible and may remove some of the important functions of schooling outlined above. An assessment has been made in Israeli kibbutzim compared to the general Israeli population to test the lowest inequality possible that removes major parts of the bias in sorting children according to parental background as well as associated attitudes and behaviors that signal those origins; in doing this, one could obtain a reference point of the lowest possible IEO possible that public policy would be able to achieve (Gilboa & Justman, 2010). One way to increase effective matching independent of parental socioeconomic status could be to increase exposure to test material in school to increase accuracy of testing and alleviate the fact that parents from socioeconomically more advantaged backgrounds are more likely to provide learning experiences through home exercises and tutoring than parents from less advantaged backgrounds. Comparative research suggests that some countries (mainly the Nordic countries, Canada, and the Netherlands) are more successful in effective matching than others (Esping-Andersen & Cimentada, 2018). Our findings suggest that lowering IEO would create substantial benefits to the cognitive functioning over the life course.

### Strengths and limitations of the study

Strength of the study was the inclusion of an extensive set of individual-level confounders. However, this research design makes the harmonization with data from other Health and Retirement Studies, such as the U.S. HRS, difficult due to incompatible measures of both outcomes and confounders. Other research designs with more variation on the country level and fewer individual covariates could provide additional insights on the links between level of IEO and cognitive functioning.

Cognitive performance of respondents at time of schooling or in early adulthood was not available, but recent research suggests that cognitive development is mainly built up through education before age 20 (Kremen et al., 2019), justifying the focus of our study on the adolescent school years of today's middle- and older-aged respondents. Other unobserved confounders contributing to educational attainment beyond cognitive ability could be gene- or environment-related factors (Stienstra et al., 2021). Indicators of early-life health and cultural capital as well as school performance were available for a subsample of the respondents, and associations consistent with expectations—higher cultural capital, better school performance, and health being associated with higher later-life cognitive functioning—suggest an important role in reducing unmeasured confounding due to early-life abilities and conditions. Childhood information assessed in the SHARELIFE surveys has been shown to be consistent with information given in earlier waves (Garrouste & Paccagnella, 2011) and quite accurate across a set of comparisons such as validation against external macro-indicators (Havari & Mazzonna, 2015). Most of the models incorporating childhood information led to similar result patterns, despite a higher share of missing data in the childhood measures due to sampling design (not all countries participated in all SHARE waves until wave 7).

Combining the life course with a cohort perspective is of particular interest due to the secular changes in educational attainment for men and women of the cohorts under investigation (Bar-Haim et al., 2019). Gender roles have also changed quite dramatically over the last century (Goldin, 2006), however, the links between IEO and gender egalitarianism are less clear, as mentioned above. Future studies with samples of geographically and culturally more diverse regions may in more detail analyze the country-cohort level associations between IEO and gender inequality.

There are many more factors relevant to educational systems that are difficult to systematically assess, such as educational quality or school segregation, that could also be relevant to explain later-life cognitive functioning (Manly et al., 2002; Walsemann et al., 2013). Thus, we cannot rule out that systematic differences in quality of education or other macro-level determinants associated with IEO may have caused the differences between the cohorts.

Historical macro-level indicators are only available for the recent decades, for example, HDI only after 1994, so a discussion on the adequacy of capturing contextual determinants aside from IEO at time of schooling of these respondents seems indicated. The use of HDI as contextual determinant possibly co-varying with levels of IEO may even be considered over controlling, as HDI is composed, among other indicators, of (average) expected and actual length of schooling, which may capture important components of IEO.

One possible limitation to a longitudinal study of older adults could be selectivity effects. Possible selectivity due to mortality in this study is probably not substantial as the sample was of younger old age and only a small fraction of respondents died over the course of the study. Since low adult socioeconomic status is linked to premature mortality, we can however not fully rule out that the exposure of interest, IEO, may have influenced mortality risk particularly of lower-educated respondents.

Further selectivity effects could come from the selected countries under investigation, as a total of five countries were missing one or two follow-up waves. Further studies are needed to test the associations of IEO and cognitive functioning in other countries and regions, and in larger samples test possible non-linear effects of level of IEO on cognitive functioning.

## Conclusions

Later-life cognitive functioning at middle and older adulthood is associated with IEO at time of schooling. Selection into education is not limited to individual skills, but also depends on educational practices that are more or less apt to match education to pupils’ cognitive skills. Understanding better the contextual determinants of cognitive functioning at younger ages and their stability during later middle age and beyond may help to design policies and interventions that aim to strengthen cognitive development and help maintain cognitive performance at older ages.

## Disclaimer

This paper uses data from the Survey of Health, Ageing and Retirement in Europe (SHARE) 2004–2017; the World Bank World Development Indicators and World Bank Global Database on Intergenerational Mobility; the UN Human Development Reports; and the WHO Global Health Observatory. The responsibility for all conclusions drawn from the data lies entirely with the authors.

Specifically, this paper uses data from SHARE Waves 1, 2, 3, 4, 5, 6, and 7 (DOIs: 10.6103/SHARE.w1.700, 10.6103/SHARE.w2.700, 10.6103/SHARE.w3.700, 10.6103/SHARE.w4.700, 10.6103/SHARE.w5.700, 10.6103/SHARE.w6.700, 10.6103/SHARE.w7.700), see Börsch-Supan et al. (2013) for methodological details. This paper additionally uses parts of Stata code published with the easySHARE data set (DOI: 10.6103/SHARE.easy.700), see Gruber et al. (2019) for methodological details.

The SHARE data collection has been funded by the European Commission through FP5 (QLK6-CT-2001-00360), FP6 (SHARE-I3: RII-CT-2006-062,193, COMPARE: CIT5-CT-2005-028,857, SHARELIFE: CIT4-CT-2006-028,812), FP7 (SHARE-PREP: GA N°211,909, SHARE-LEAP: GA N°227,822, SHARE M4: GA N°261,982), and Horizon 2020 (SHARE-DEV3: GA N°676,536, SERISS: GA N°654,221) and by DG Employment, Social Affairs & Inclusion. Additional funding from the German Ministry of Education and Research, the Max Planck Society for the Advancement of Science, the U.S. National Institute on Aging (U01_AG09740-13S2, P01_AG005842, P01_AG08291, P30_AG12815, R21_AG025169, Y1-AG-4553-01, IAG_BSR06-11, OGHA_04–064, HHSN271201300071C), and from various national funding sources is gratefully acknowledged (see www.share-project.org).

## Financial disclosure statement/funding details

This work was supported by the 10.13039/100010663European Research Council (grant agreement no. 803239, 2019–2023, to AKL); and the National Research Fund Luxembourg (FNR/P11/05 and FNR/P11/05 bis, 2012–2018, to LC).

## Author contributions

AKL is lead investigator, she set up the study design, analyzed the data and wrote the draft. EBH contributed to study design and data analysis. LC and EBH suggested additional analyses and revised the draft for intellectual content.

## Declaration of competing interest

The authors declare no conflict of interest.
