# Supplementary material for: Inequality of educational opportunity at time of schooling predicts cognitive functioning in later adulthood
Source: SSM Popul Health. 2021 Jun 5;15:100837. doi: 10.1016/j.ssmph.2021.100837 (PMC8193135; doi:10.1016/j.ssmph.2021.100837)
Supplement: Multimedia component 1 [file mmc1.docx]

**Supplemental Material**

**Inequality of Educational Opportunity at Time of Schooling Predicts Cognitive Functioning in Later Adulthood**

- Appendix Figure 1: Sample eligibility flowchart
- Appendix Figure 2: Unadjusted female advantage in cognitive performance by IEO.
- Appendix Table 1. Associations of IEO on level of and rate of change in three cognitive measures in stratified multilevel (mixed-effects) models in the subsample with childhood information.
- Appendix Table 2: Results from the mixed-effects models additionally controlling for either GDP, HLE or HDI.
- Appendix Table 3: Results from the mixed-effects models additionally controlling for occupational level (ISCO) of current or last job.
- Appendix Table 4. Interactions of IEO, education, and gender in mixed-effects models.


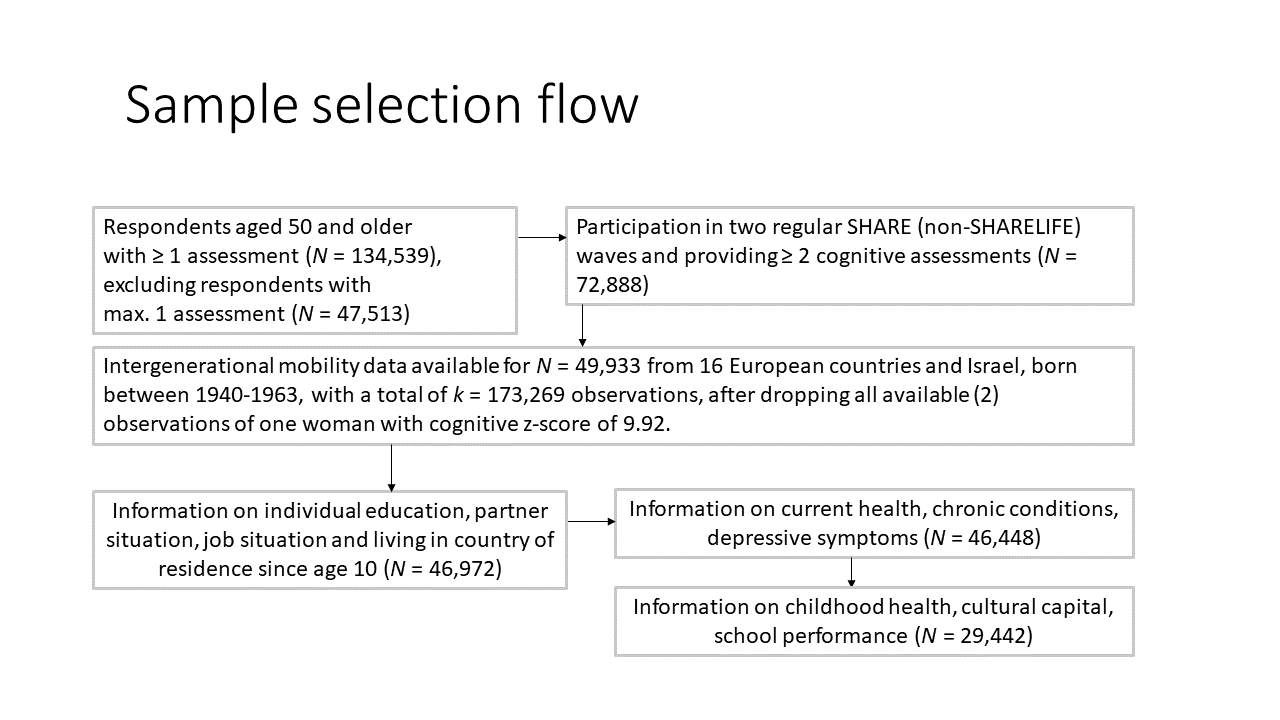


Appendix Figure 1: Sample eligibility flowchart.

Note. Although SHARE provides data of a total of N = 139,566 individuals who participated in ≥1 wave, not all are age-eligible. Participants with at least two and up to six cognitive measurements were included. This led to the exclusion of a total of 47,513 respondents. Two of the seven SHARE waves were SHARELIFE interviews in which no health assessment (including cognitive) took place. One country, Luxembourg, was not available in the GDIM dataset of May 2018, which led to the exclusion of an additional 1,732 respondents.

Appendix Figure 2: Unadjusted female advantage in cognitive performance by IEO.

Note: The y axis represents the difference between female and male scores on the cognitive composite score (average of the three z-standardized cognitive performance measures at first assessment). The size of the data points reflects the sample sizes (45 ≤ *n* ≤ 2,195, see methods).

| Appendix Table 1. Associations of IEO on level of and rate of change in three cognitive measures in stratified multilevel (mixed-effects) models in the subsample with childhood information. | | | | | | |
| --- | --- | --- | --- | --- | --- | --- |
|  | **Women** |  |  | **Men** |  |  |
|  | **Immediate recall** | **Delayed recall** | **Verbal fluency** | **Immediate recall** | **Delayed recall** | **Verbal fluency** |
|  | Coeff. (CI) | Coeff. (CI) | Coeff. (CI) | Coeff. (CI) | Coeff. (CI) | Coeff. (CI) |
| (Intercept) | -0.03  (-0.14 – 0.08) | 0.04  (-0.07 – 0.16) | -0.09  (-0.22 – 0.04) | -0.02  (-0.13 – 0.09) | 0.05  (-0.07 – 0.17) | -0.11  (-0.25 – 0.02) |
| Age centered at 50 in decades | 0.07 ^*^ (0.01 – 0.13) | 0.05  (-0.01 – 0.11) | 0.08 ^*^ (0.01 – 0.15) | 0.06  (-0.01 – 0.12) | 0.04  (-0.03 – 0.11) | -0.00  (-0.08 – 0.08) |
| Age squared | -0.09 ^***^ (-0.11 – -0.07) | -0.10 ^***^ (-0.12 – -0.08) | -0.09 ^***^ (-0.11 – -0.07) | -0.09 ^***^ (-0.11 – -0.07) | -0.09 ^***^ (-0.11 – -0.06) | -0.05 ^***^ (-0.07 – -0.02) |
| Indicator of first testing | -0.09 ^***^ (-0.11 – -0.07) | -0.15 ^***^ (-0.16 – -0.13) | -0.06 ^***^ (-0.07 – -0.04) | -0.07 ^***^ (-0.09 – -0.05) | -0.12 ^***^ (-0.14 – -0.10) | -0.06 ^***^ (-0.08 – -0.04) |
| Education (reference: ISCED 0-2) |  |  |  |  |  |  |
| ISCED 3 | 0.27 ^***^ (0.24 – 0.30) | 0.24 ^***^ (0.22 – 0.27) | 0.23 ^***^ (0.20 – 0.26) | 0.20 ^***^ (0.17 – 0.24) | 0.17 ^***^ (0.14 – 0.21) | 0.15 ^***^ (0.12 – 0.19) |
| ISCED 4-6 | 0.43 ^***^ (0.40 – 0.46) | 0.41 ^***^ (0.38 – 0.45) | 0.43 ^***^ (0.39 – 0.46) | 0.38 ^***^ (0.35 – 0.42) | 0.37 ^***^ (0.34 – 0.41) | 0.29 ^***^ (0.25 – 0.33) |
| Job situation (reference: Retired) |  |  |  |  |  |  |
| Employed or self-employed | 0.08 ^***^ (0.05 – 0.11) | 0.09 ^***^ (0.05 – 0.12) | 0.06 ^***^ (0.03 – 0.10) | 0.07 ^***^ (0.03 – 0.10) | 0.07 ^***^ (0.04 – 0.11) | 0.07 ^***^ (0.03 – 0.11) |
| Unemployed | 0.01  (-0.05 – 0.06) | 0.00  (-0.06 – 0.06) | -0.04  (-0.10 – 0.02) | -0.05  (-0.11 – 0.00) | -0.04  (-0.10 – 0.03) | -0.08 ^*^ (-0.14 – -0.02) |
| Permanently sick or disabled | -0.12 ^***^ (-0.18 – -0.06) | -0.10 ^**^ (-0.17 – -0.04) | -0.10 ^**^ (-0.16 – -0.03) | -0.15 ^***^ (-0.21 – -0.09) | -0.12 ^***^ (-0.19 – -0.06) | -0.15 ^***^ (-0.22 – -0.08) |
| Homemaker | -0.05 ^*^ (-0.09 – -0.01) | -0.02  (-0.06 – 0.02) | -0.07 ^***^ (-0.11 – -0.03) | 0.03  (-0.16 – 0.22) | 0.10  (-0.10 – 0.31) | -0.08  (-0.29 – 0.13) |
| Cohabitation status (reference: Living alone) |  |  |  |  |  |  |
| Living with partner | 0.03 ^**^ (0.01 – 0.06) | -0.01  (-0.03 – 0.02) | 0.05 ^***^ (0.03 – 0.08) | 0.08 ^***^ (0.05 – 0.11) | 0.07 ^***^ (0.03 – 0.10) | 0.09 ^***^ (0.06 – 0.13) |
| Self-rated health (reference: fair or better) |  |  |  |  |  |  |
| Poor | -0.17 ^***^ (-0.21 – -0.13) | -0.17 ^***^ (-0.21 – -0.12) | -0.17 ^***^ (-0.21 – -0.13) | -0.19 ^***^ (-0.23 – -0.14) | -0.19 ^***^ (-0.24 – -0.14) | -0.15 ^***^ (-0.21 – -0.10) |
| Number of chronic conditions | -0.02 ^***^ (-0.03 – -0.01) | -0.02 ^***^ (-0.03 – -0.01) | -0.02 ^***^ (-0.03 – -0.01) | 0.00  (-0.01 – 0.01) | -0.01 ^*^ (-0.02 – -0.00) | 0.00  (-0.01 – 0.02) |
| Number of depressive symptoms | -0.02 ^***^ (-0.02 – -0.01) | -0.02 ^***^ (-0.02 – -0.01) | -0.02 ^***^ (-0.03 – -0.02) | -0.03 ^***^ (-0.03 – -0.02) | -0.03 ^***^ (-0.03 – -0.02) | -0.03 ^***^ (-0.04 – -0.02) |
| Childhood information (retrospective) |  |  |  |  |  |  |
| No. of books at age 10 (reference: 0-10 books) |  |  |  |  |  |  |
| Enough to fill one shelf (11-25 books) | 0.13 ^***^ (0.10 – 0.16) | 0.15 ^***^ (0.12 – 0.18) | 0.13 ^***^ (0.10 – 0.16) | 0.15 ^***^ (0.12 – 0.18) | 0.16 ^***^ (0.12 – 0.19) | 0.13 ^***^ (0.09 – 0.17) |
| Enough to fill one bookcase (26-100 books) | 0.20 ^***^ (0.17 – 0.23) | 0.23 ^***^ (0.20 – 0.26) | 0.23 ^***^ (0.20 – 0.26) | 0.24 ^***^ (0.20 – 0.27) | 0.26 ^***^ (0.22 – 0.29) | 0.27 ^***^ (0.23 – 0.30) |
| Enough to fill two bookcases (101-200 books) | 0.25 ^***^ (0.21 – 0.29) | 0.31 ^***^ (0.27 – 0.35) | 0.32 ^***^ (0.28 – 0.36) | 0.28 ^***^ (0.24 – 0.33) | 0.29 ^***^ (0.24 – 0.33) | 0.37 ^***^ (0.32 – 0.42) |
| Enough to fill two or more bookcases (more than 200) | 0.30 ^***^ (0.26 – 0.34) | 0.36 ^***^ (0.32 – 0.41) | 0.39 ^***^ (0.35 – 0.44) | 0.34 ^***^ (0.29 – 0.38) | 0.35 ^***^ (0.31 – 0.40) | 0.45 ^***^ (0.40 – 0.50) |
| School performance math compared to peers (reference: Much better) |  |  |  |  |  |  |
| Better | 0.03  (-0.02 – 0.08) | 0.01  (-0.05 – 0.07) | 0.00  (-0.05 – 0.06) | -0.04  (-0.10 – 0.01) | -0.04  (-0.09 – 0.02) | 0.00  (-0.06 – 0.06) |
| About the same | -0.06 ^*^ (-0.11 – -0.01) | -0.09 ^**^ (-0.14 – -0.03) | -0.08 ^**^ (-0.13 – -0.02) | -0.13 ^***^ (-0.18 – -0.08) | -0.14 ^***^ (-0.19 – -0.09) | -0.08 ^**^ (-0.13 – -0.02) |
| Worse | -0.16 ^***^ (-0.21 – -0.11) | -0.19 ^***^ (-0.25 – -0.13) | -0.19 ^***^ (-0.25 – -0.13) | -0.22 ^***^ (-0.27 – -0.16) | -0.24 ^***^ (-0.30 – -0.18) | -0.18 ^***^ (-0.25 – -0.12) |
| Much worse | -0.22 ^***^ (-0.30 – -0.15) | -0.26 ^***^ (-0.34 – -0.17) | -0.22 ^***^ (-0.31 – -0.14) | -0.35 ^***^ (-0.44 – -0.26) | -0.37 ^***^ (-0.47 – -0.27) | -0.22 ^***^ (-0.32 – -0.12) |
| School performance language compared to peers (reference: Much better) |  |  |  |  |  |  |
| Better | -0.08 ^**^ (-0.12 – -0.03) | -0.04  (-0.09 – 0.01) | -0.07 ^**^ (-0.11 – -0.02) | -0.01  (-0.07 – 0.06) | -0.03  (-0.10 – 0.04) | 0.00  (-0.07 – 0.07) |
| About the same | -0.17 ^***^ (-0.22 – -0.13) | -0.14 ^***^ (-0.19 – -0.09) | -0.16 ^***^ (-0.21 – -0.11) | -0.13 ^***^ (-0.19 – -0.07) | -0.12 ^***^ (-0.19 – -0.05) | -0.11 ^**^ (-0.18 – -0.04) |
| Worse | -0.26 ^***^ (-0.31 – -0.20) | -0.19 ^***^ (-0.25 – -0.14) | -0.18 ^***^ (-0.24 – -0.12) | -0.22 ^***^ (-0.29 – -0.15) | -0.18 ^***^ (-0.25 – -0.11) | -0.12 ^**^ (-0.19 – -0.05) |
| Much worse | -0.38 ^***^ (-0.48 – -0.28) | -0.30 ^***^ (-0.41 – -0.19) | -0.27 ^***^ (-0.38 – -0.16) | -0.20 ^***^ (-0.30 – -0.10) | -0.19 ^***^ (-0.29 – -0.08) | -0.08  (-0.20 – 0.03) |
| Self-rated health in childhood (reference: Excellent) |  |  |  |  |  |  |
| Very good | 0.01  (-0.02 – 0.04) | 0.02  (-0.01 – 0.05) | -0.01  (-0.04 – 0.02) | 0.01  (-0.02 – 0.04) | 0.03  (-0.00 – 0.06) | -0.02  (-0.06 – 0.01) |
| Good | -0.05 ^***^ (-0.08 – -0.02) | -0.02  (-0.05 – 0.02) | -0.05 ^**^ (-0.08 – -0.02) | -0.03 ^*^ (-0.06 – -0.00) | -0.03  (-0.06 – 0.01) | -0.08 ^***^ (-0.11 – -0.04) |
| Fair | -0.01  (-0.05 – 0.03) | -0.02  (-0.06 – 0.02) | -0.05 ^**^ (-0.09 – -0.01) | -0.03  (-0.07 – 0.01) | -0.02  (-0.06 – 0.03) | -0.08 ^**^ (-0.13 – -0.03) |
| Poor | -0.01  (-0.07 – 0.04) | -0.03  (-0.09 – 0.03) | -0.04  (-0.09 – 0.02) | -0.01  (-0.08 – 0.05) | -0.01  (-0.08 – 0.06) | -0.05  (-0.12 – 0.02) |
| IEO | -0.97 ^*^ (-1.87 – -0.06) | -1.14 ^*^ (-2.14 – -0.14) | -1.85 ^**^ (-2.99 – -0.72) | -0.78 ^*^ (-1.53 – -0.02) | -0.90 ^*^ (-1.71 – -0.09) | -2.05 ^***^ (-3.07 – -1.02) |
| IEO*age | 0.08  (-0.18 – 0.33) | 0.22  (-0.03 – 0.48) | -0.10  (-0.39 – 0.20) | 0.53 ^***^ (0.25 – 0.80) | 0.46 ^**^ (0.18 – 0.75) | 0.27  (-0.07 – 0.61) |
| **Random Effects** | | | | | | |
| σ^2^ | 0.55 | 0.55 | 0.42 | 0.58 | 0.57 | 0.46 |
| τ_00 individual-level_ | 0.24 | 0.31 | 0.31 | 0.24 | 0.31 | 0.33 |
| τ_00 country-cohort level_ | 0.07 | 0.08 | 0.11 | 0.04 | 0.05 | 0.08 |
| ICC | 0.36 | 0.42 | 0.50 | 0.33 | 0.38 | 0.47 |
| N | 16,261 | 16,261 | 16,257 | 13,181 | 13,181 | 13,178 |
| Number of country-cohorts | 49 | 49 | 49 | 49 | 49 | 49 |
| Observations | 53,365 | 53,383 | 41,039 | 42,098 | 42,142 | 32,541 |
| Marginal R^2^ / Conditional R^2^ | 0.159 / 0.458 | 0.160 / 0.509 | 0.191 / 0.596 | 0.131 / 0.415 | 0.129 / 0.460 | 0.141 / 0.546 |
| *Note. * p*<0.05.*** p*<0.01*. *** p*<0.001. std., standardized, CI, confidence interval. ISCED, International Standard Classification of Education, No., number, IEO, Inequality of educational opportunity, σ^2^ , mean random effect variance (overall), τ_00_, random effect (between-units) variances on individual level and country-cohort level, ICC, Intra-class correlation, N, number of respondents, Marginal R^2^, variance explained by fixed effects, Conditional R^2^, variance explained by fixed and random effects. | | | | | | |

| Appendix Table 2. Results from the mixed-effects models in stratified analyses, additionally controlling for occupational level of current or last job. | | | | | | |
| --- | --- | --- | --- | --- | --- | --- |
|  | **Women** |  |  | **Men** |  |  |
|  | **Immediate recall** | **Delayed recall** | **Verbal fluency** | **Immediate recall** | **Delayed recall** | **Verbal fluency** |
|  | Coeff. (CI) | Coeff. (CI) | Coeff. (CI) | Coeff. (CI) | Coeff. (CI) | Coeff. (CI) |
| (Intercept) | 0.04  (-0.07 – 0.16) | 0.16 ^**^ (0.04 – 0.28) | -0.01  (-0.14 – 0.12) | 0.03  (-0.07 – 0.14) | 0.09  (-0.02 – 0.20) | -0.01  (-0.14 – 0.11) |
| Age centered at 50 in decades | 0.10 ^*^ (0.01 – 0.19) | 0.07  (-0.02 – 0.16) | 0.07  (-0.02 – 0.16) | 0.10 ^*^ (0.01 – 0.20) | 0.10 ^*^ (0.00 – 0.20) | 0.03  (-0.07 – 0.13) |
| Age squared | -0.08 ^***^ (-0.11 – -0.04) | -0.07 ^***^ (-0.11 – -0.03) | -0.06 ^***^ (-0.10 – -0.03) | -0.08 ^***^ (-0.12 – -0.04) | -0.06 ^**^ (-0.10 – -0.02) | -0.04  (-0.08 – 0.00) |
| Indicator of first testing | -0.14 ^***^ (-0.18 – -0.09) | -0.19 ^***^ (-0.24 – -0.15) | -0.08 ^***^ (-0.12 – -0.04) | -0.07 ^**^ (-0.11 – -0.02) | -0.15 ^***^ (-0.20 – -0.11) | -0.06 ^**^ (-0.10 – -0.02) |
| Education (reference: ISCED 0-2) |  |  |  |  |  |  |
| ISCED 3 | 0.27 ^***^ (0.23 – 0.30) | 0.22 ^***^ (0.19 – 0.26) | 0.22 ^***^ (0.19 – 0.25) | 0.26 ^***^ (0.22 – 0.30) | 0.22 ^***^ (0.19 – 0.26) | 0.18 ^***^ (0.14 – 0.21) |
| ISCED 4-6 | 0.44 ^***^ (0.40 – 0.47) | 0.39 ^***^ (0.35 – 0.43) | 0.46 ^***^ (0.42 – 0.50) | 0.40 ^***^ (0.36 – 0.44) | 0.38 ^***^ (0.34 – 0.43) | 0.33 ^***^ (0.28 – 0.37) |
| Job situation (reference: Retired) |  |  |  |  |  |  |
| Employed or self-employed | 0.09 ^***^ (0.06 – 0.13) | 0.11 ^***^ (0.07 – 0.15) | 0.09 ^***^ (0.05 – 0.13) | 0.07 ^***^ (0.03 – 0.11) | 0.10 ^***^ (0.06 – 0.14) | 0.07 ^***^ (0.03 – 0.12) |
| Unemployed | 0.02  (-0.04 – 0.08) | -0.01  (-0.07 – 0.05) | -0.03  (-0.09 – 0.02) | -0.03  (-0.09 – 0.03) | -0.02  (-0.08 – 0.04) | -0.03  (-0.09 – 0.03) |
| Permanently sick or disabled | -0.10 ^**^ (-0.16 – -0.03) | -0.06  (-0.13 – 0.01) | -0.04  (-0.11 – 0.02) | -0.10 ^**^ (-0.17 – -0.03) | -0.08 ^*^ (-0.15 – -0.00) | -0.10 ^**^ (-0.17 – -0.03) |
| Homemaker | 0.04  (-0.00 – 0.09) | 0.04  (-0.01 – 0.09) | 0.06 ^*^ (0.01 – 0.11) | 0.06  (-0.17 – 0.30) | 0.07  (-0.17 – 0.31) | 0.17  (-0.06 – 0.40) |
| Cohabitation status (reference: Living alone) |  |  |  |  |  |  |
| Living with partner | 0.04 ^**^ (0.01 – 0.06) | 0.01  (-0.02 – 0.04) | 0.04 ^**^ (0.02 – 0.07) | 0.05 ^**^ (0.02 – 0.09) | 0.04 ^*^ (0.01 – 0.08) | 0.08 ^***^ (0.04 – 0.11) |
| Self-rated health (reference: Fair or better) |  |  |  |  |  |  |
| Poor | -0.14 ^***^ (-0.19 – -0.09) | -0.11 ^***^ (-0.16 – -0.06) | -0.13 ^***^ (-0.18 – -0.08) | -0.15 ^***^ (-0.21 – -0.10) | -0.12 ^***^ (-0.18 – -0.06) | -0.12 ^***^ (-0.18 – -0.07) |
| Number of chronic conditions | -0.01 ^*^ (-0.03 – -0.00) | -0.01  (-0.02 – 0.00) | -0.01  (-0.02 – 0.00) | -0.00  (-0.01 – 0.01) | -0.00  (-0.02 – 0.01) | 0.01  (-0.00 – 0.02) |
| Number of depressive symptoms | -0.02 ^***^ (-0.03 – -0.02) | -0.03 ^***^ (-0.03 – -0.02) | -0.02 ^***^ (-0.03 – -0.02) | -0.04 ^***^ (-0.05 – -0.03) | -0.05 ^***^ (-0.05 – -0.04) | -0.03 ^***^ (-0.04 – -0.02) |
| Professional | 0.05  (-0.01 – 0.11) | 0.07 ^*^ (0.01 – 0.14) | 0.01  (-0.05 – 0.07) | 0.02  (-0.03 – 0.08) | 0.08 ^**^ (0.02 – 0.14) | 0.00  (-0.05 – 0.06) |
| Technician | -0.01  (-0.07 – 0.05) | -0.00  (-0.07 – 0.06) | -0.08 ^**^ (-0.14 – -0.02) | -0.09 ^***^ (-0.14 – -0.04) | -0.06 ^*^ (-0.12 – -0.01) | -0.08 ^**^ (-0.13 – -0.02) |
| Clerk | -0.04  (-0.10 – 0.01) | -0.02  (-0.08 – 0.04) | -0.09 ^***^ (-0.15 – -0.04) | -0.08 ^**^ (-0.14 – -0.02) | -0.04  (-0.10 – 0.02) | -0.15 ^***^ (-0.21 – -0.10) |
| Services, sales worker | -0.16 ^***^ (-0.22 – -0.11) | -0.15 ^***^ (-0.21 – -0.09) | -0.19 ^***^ (-0.25 – -0.14) | -0.17 ^***^ (-0.22 – -0.11) | -0.12 ^***^ (-0.17 – -0.06) | -0.18 ^***^ (-0.24 – -0.13) |
| Skilled agricultural, fishery worker | -0.28 ^***^ (-0.36 – -0.19) | -0.28 ^***^ (-0.37 – -0.20) | -0.31 ^***^ (-0.39 – -0.23) | -0.30 ^***^ (-0.38 – -0.23) | -0.32 ^***^ (-0.39 – -0.24) | -0.23 ^***^ (-0.30 – -0.16) |
| Craft, trades worker | -0.25 ^***^ (-0.32 – -0.18) | -0.23 ^***^ (-0.31 – -0.16) | -0.28 ^***^ (-0.35 – -0.21) | -0.34 ^***^ (-0.39 – -0.29) | -0.27 ^***^ (-0.33 – -0.22) | -0.28 ^***^ (-0.34 – -0.23) |
| Plant, machine operator, assembler | -0.31 ^***^ (-0.39 – -0.23) | -0.35 ^***^ (-0.43 – -0.26) | -0.38 ^***^ (-0.46 – -0.30) | -0.33 ^***^ (-0.39 – -0.27) | -0.31 ^***^ (-0.37 – -0.25) | -0.32 ^***^ (-0.38 – -0.26) |
| Elementary occupation | -0.33 ^***^ (-0.39 – -0.27) | -0.33 ^***^ (-0.40 – -0.27) | -0.35 ^***^ (-0.41 – -0.29) | -0.41 ^***^ (-0.47 – -0.35) | -0.33 ^***^ (-0.39 – -0.27) | -0.42 ^***^ (-0.48 – -0.36) |
| Armed forces | 0.09  (-0.11 – 0.29) | -0.01  (-0.22 – 0.20) | -0.12  (-0.31 – 0.07) | -0.09  (-0.22 – 0.04) | -0.05  (-0.18 – 0.09) | -0.11  (-0.24 – 0.02) |
| IEO | -1.30 ^**^ (-2.04 – -0.56) | -1.48 ^***^ (-2.27 – -0.70) | -2.05 ^***^ (-3.18 – -0.92) | -1.13 ^***^ (-1.77 – -0.50) | -1.10 ^***^ (-1.68 – -0.52) | -2.32 ^***^ (-3.31 – -1.33) |
| IEO*age | 0.36  (-0.02 – 0.74) | 0.57 ^**^ (0.18 – 0.96) | -0.17  (-0.55 – 0.20) | 0.79 ^***^ (0.40 – 1.18) | 0.65 ^**^ (0.26 – 1.03) | 0.29  (-0.11 – 0.69) |
| **Random Effects** | | | | | | |
| σ^2^ | 0.56 | 0.54 | 0.39 | 0.55 | 0.53 | 0.46 |
| τ_00 individual-level_ | 0.23 | 0.29 | 0.35 | 0.28 | 0.34 | 0.33 |
| τ_00 country-cohort_ | 0.04 | 0.04 | 0.11 | 0.02 | 0.02 | 0.08 |
| ICC | 0.32 | 0.38 | 0.54 | 0.36 | 0.40 | 0.47 |
| N | 21,046 | 21,039 | 21,023 | 17,888 | 17,885 | 17,857 |
| Number of country-cohorts | 49 | 49 | 49 | 49 | 49 | 49 |
| Observations | 22,397 | 22,391 | 22,372 | 19,178 | 19,173 | 19,144 |
| Marginal R^2^ / Conditional R^2^ | 0.131 / 0.412 | 0.126 / 0.457 | 0.160 / 0.617 | 0.114 / 0.433 | 0.109 / 0.467 | 0.123 / 0.537 |
| Note. ** p*<0.05.*** p*<0.01*. *** p*<0.001. std., standardized, CI, confidence interval, ISCED, International Standard Classification of Education, No., number, IEO, Inequality of educational opportunity, σ^2^ , mean random effect variance (overall), τ_00_, random effect (between-units) variances on individual level and country-cohort level, ICC, Intra-class correlation, N, number of respondents, Marginal R^2^, variance explained by fixed effects, Conditional R^2^, variance explained by fixed and random effects. Adjusted for age, age squared, indicator of first testing, current job situation, cohabitation status. Level of occupation was categorized with one-digit International Standard Classification of Occupation (ISCO-08) structure.   \| Appendix Table. Main coefficients and model statistics from the mixed-effects models that additionally control for (1) GDP, (2) HLE, and (3) HDI. \| \| \| \| \| \| \| \| --- \| --- \| --- \| --- \| --- \| --- \| --- \| \|  \| **Women** \|  \|  \| **Men** \|  \|  \| \|  \| **Immediate recall** \| **Delayed recall** \| **Verbal fluency** \| **Immediate recall** \| **Delayed recall** \| **Verbal fluency** \| \|  \| Coeff. (CI) \| Coeff. (CI) \| Coeff. (CI) \| Coeff. (CI) \| Coeff. (CI) \| Coeff. (CI) \| \| (1) Main model (Table 2) additionally controlling for GDP \| \| \| \| \| \| \| \| GDP \| 0.10 ^***^ (0.05 – 0.16) \| 0.16 ^***^ (0.11 – 0.21) \| 0.07  (-0.02 – 0.16) \| 0.07 ^**^ (0.03 – 0.11) \| 0.11 ^***^ (0.08 – 0.15) \| 0.05  (-0.02 – 0.13) \| \| IEO \| -1.14 ^**^ (-1.80 – -0.49) \| -0.84 ^*^ (-1.46 – -0.22) \| -1.70 ^**^ (-2.75 – -0.66) \| -0.89 ^**^ (-1.40 – -0.38) \| -0.54 ^*^ (-0.99 – -0.08) \| -1.74 ^***^ (-2.68 – -0.81) \| \| IEO*age \| 0.16 ^*^ (0.01 – 0.31) \| -0.18 ^*^ (-0.33 – -0.03) \| -0.39 ^***^ (-0.54 – -0.25) \| 0.47 ^***^ (0.30 – 0.63) \| -0.00  (-0.17 – 0.17) \| -0.16  (-0.33 – 0.01) \| \| Random effects \|  \|  \|  \|  \|  \|  \| \| σ^2^ \| 0.55 \| 0.55 \| 0.41 \| 0.58 \| 0.57 \| 0.45 \| \| τ_00 individual-level_ \| 0.26 \| 0.32 \| 0.32 \| 0.27 \| 0.32 \| 0.35 \| \| τ_00 country-cohort_ \| 0.04 \| 0.03 \| 0.10 \| 0.02 \| 0.01 \| 0.08 \| \| ICC \| 0.35 \| 0.39 \| 0.51 \| 0.33 \| 0.37 \| 0.49 \| \| N \| 25,544 \| 25,544 \| 25,538 \| 20,904 \| 20,903 \| 20,900 \| \| Number of country-cohorts \| 49 \| 49 \| 49 \| 49 \| 49 \| 49 \| \| Observations \| 89,713 \| 89,748 \| 77,284 \| 71,455 \| 71,525 \| 61,807 \| \| Marginal R^2^ / Conditional R^2^ \| 0.144 / 0.441 \| 0.151 / 0.483 \| 0.177 / 0.596 \| 0.107 / 0.402 \| 0.113 / 0.443 \| 0.125 / 0.554 \| \| (2) Main model (Table 2) additionally controlling for HLE \| \| \| \| \| \| \| \| HLE \| 0.01  (-0.04 – 0.07) \| 0.07 ^*^ (0.02 – 0.12) \| -0.06  (-0.13 – 0.01) \| 0.00  (-0.04 – 0.04) \| 0.05 ^*^ (0.01 – 0.09) \| -0.05  (-0.11 – 0.02) \| \| IEO \| -1.30 ^**^ (-2.08 – -0.51) \| -1.32 ^**^ (-2.13 – -0.51) \| -1.47 ^*^ (-2.58 – -0.37) \| -0.95 ^**^ (-1.54 – -0.35) \| -0.86 ^**^ (-1.46 – -0.26) \| -1.55 ^**^ (-2.54 – -0.56) \| \| IEO*age \| 0.17 ^*^ (0.02 – 0.31) \| -0.17 ^*^ (-0.33 – -0.02) \| -0.39 ^***^ (-0.53 – -0.24) \| 0.48 ^***^ (0.32 – 0.65) \| 0.01  (-0.16 – 0.17) \| -0.16  (-0.32 – 0.01) \| \| Random effects \|  \|  \|  \|  \|  \|  \| \| σ^2^ \| 0.55 \| 0.55 \| 0.41 \| 0.58 \| 0.57 \| 0.45 \| \| τ_00 individual-level_ \| 0.26 \| 0.32 \| 0.32 \| 0.27 \| 0.32 \| 0.35 \| \| τ_00 country-cohort_ \| 0.05 \| 0.05 \| 0.10 \| 0.02 \| 0.02 \| 0.08 \| \| ICC \| 0.36 \| 0.40 \| 0.51 \| 0.33 \| 0.38 \| 0.49 \| \| N \| 25,544 \| 25,544 \| 25,538 \| 20,904 \| 20,903 \| 20,900 \| \| Number of country-cohorts \| 49 \| 49 \| 49 \| 49 \| 49 \| 49 \| \| Observations \| 89,713 \| 89,748 \| 77,284 \| 71,455 \| 71,525 \| 61,807 \| \| Marginal R^2^ / Conditional R^2^ \| 0.130 / 0.440 \| 0.131 / 0.483 \| 0.180 / 0.597 \| 0.097 / 0.398 \| 0.098 / 0.441 \| 0.121 / 0.552 \| \| (3) Main model (Table 2) additionally controlling for HDI \| \| \| \| \| \| \| \| HDI \| 3.34 ^**^ (1.42 – 5.26) \| 4.72 ^***^ (2.78 – 6.66) \| 3.12 ^*^ (0.11 – 6.13) \| 2.35 ^**^ (0.93 – 3.77) \| 3.58 ^***^ (2.19 – 4.97) \| 2.76 ^*^ (0.10 – 5.42) \| \| IEO \| -0.77 ^*^ (-1.49 – -0.05) \| -0.33  (-1.05 – 0.40) \| -1.34 ^*^ (-2.45 – -0.23) \| -0.62 ^*^ (-1.17 – -0.07) \| -0.17  (-0.71 – 0.37) \| -1.42 ^**^ (-2.40 – -0.43) \| \| IEO*age \| 0.16 ^*^ (0.01 – 0.31) \| -0.17 ^*^ (-0.33 – -0.02) \| -0.39 ^***^ (-0.54 – -0.25) \| 0.47 ^***^ (0.31 – 0.64) \| 0.04  (-0.13 – 0.22) \| -0.16  (-0.33 – 0.01) \| \| Random effects \|  \|  \|  \|  \|  \|  \| \| σ2 \| 0.55 \| 0.55 \| 0.41 \| 0.58 \| 0.55 \| 0.45 \| \| τ00 individual-level \| 0.26 \| 0.32 \| 0.32 \| 0.27 \| 0.42 \| 0.35 \| \| τ00 country-cohort \| 0.04 \| 0.04 \| 0.10 \| 0.02 \| 0.02 \| 0.07 \| \| ICC \| 0.35 \| 0.40 \| 0.51 \| 0.33 \| 0.40 \| 0.49 \| \| N \| 25,544 \| 25,544 \| 25,538 \| 20,904 \| 20,903 \| 20,900 \| \| Number of country-cohorts \| 49 \| 49 \| 49 \| 49 \| 49 \| 49 \| \| Observations \| 89,713 \| 89,748 \| 77,284 \| 71,455 \| 71,525 \| 61,807 \| \| Marginal R2 / Conditional R2 \| 0.144 / 0.443 \| 0.146 / 0.485 \| 0.182 / 0.597 \| 0.107 / 0.402 \| 0.108 / 0.462 \| 0.130 / 0.555 \|   *Note*. ** p*<0.05.*** p*<0.01*. *** p*<0.001. std., standardized, CI, confidence interval, GDP, Gross Domestic Product, IEO, Inequality of educational opportunity, σ^2^ , mean random effect variance (overall), τ_00_, random effect (between-units) variances on individual level and country-cohort level, ICC, Intra-class correlation, N, number of respondents, Marginal R^2^, variance explained by fixed effects, Conditional R^2^, variance explained by fixed and random effects, HLE, Healthy life expectancy at age 60, HDI, Human Development Index. Adjusted for age, age squared, indicator of first testing, current job situation, cohabitation status, education, current job situation, reporting poor health, number of chronic conditions, and number of depressive symptoms (coefficients not shown). | | | | | | |

| Appendix Table 4. Interactions of IEO, education, and gender in mixed-effects models. | | | |  |
| --- | --- | --- | --- | --- |
|  | **Immediate recall** | **Delayed recall** | **Verbal fluency** | |
|  | Coeff. (CI) | Coeff. (CI) | Coeff. (CI) | |
| (Intercept) | -0.40 ^***^ (-0.47 – -0.34) | -0.35 ^***^ (-0.42 – -0.28) | -0.27 ^***^ (-0.36 – -0.18) | |
| Age centered at 50 in decades | 0.16 ^***^ (0.13 – 0.19) | 0.18 ^***^ (0.15 – 0.21) | 0.06 ^***^ (0.03 – 0.09) | |
| Age squared | -0.10 ^***^ (-0.11 – -0.09) | -0.11 ^***^ (-0.12 – -0.10) | -0.06 ^***^ (-0.07 – -0.05) | |
| Indicator of first testing | -0.07 ^***^ (-0.08 – -0.06) | -0.11 ^***^ (-0.12 – -0.10) | -0.05 ^***^ (-0.06 – -0.04) | |
| Gender (reference: Male) |  |  |  | |
| Female | 0.22 ^***^ (0.19 – 0.24) | 0.22 ^***^ (0.20 – 0.25) | -0.00  (-0.03 – 0.02) | |
| Education (reference: ISCED 0-2) |  |  |  | |
| ISCED 3 | 0.29 ^***^ (0.26 – 0.31) | 0.25 ^***^ (0.22 – 0.27) | 0.23 ^***^ (0.21 – 0.26) | |
| ISCED 4-6 | 0.55 ^***^ (0.52 – 0.57) | 0.51 ^***^ (0.49 – 0.54) | 0.49 ^***^ (0.46 – 0.51) | |
| Current situation (reference: Retired) |  |  |  | |
| Employed or self-employed | 0.10 ^***^ (0.08 – 0.12) | 0.11 ^***^ (0.09 – 0.13) | 0.09 ^***^ (0.07 – 0.11) | |
| Unemployed | -0.02  (-0.05 – 0.01) | -0.01  (-0.05 – 0.02) | -0.07 ^***^ (-0.11 – -0.04) | |
| Permanently sick or disabled | -0.13 ^***^ (-0.16 – -0.09) | -0.10 ^***^ (-0.13 – -0.06) | -0.15 ^***^ (-0.18 – -0.11) | |
| Homemaker | -0.04 ^**^ (-0.06 – -0.01) | -0.02  (-0.05 – 0.00) | -0.06 ^***^ (-0.09 – -0.03) | |
| Cohabitation status (reference: Living alone) |  |  |  | |
| Living with partner | 0.05 ^***^ (0.04 – 0.07) | 0.03 ^**^ (0.01 – 0.04) | 0.06 ^***^ (0.05 – 0.08) | |
| Self-rated health (reference: Fair or better) |  |  |  | |
| Poor | -0.18 ^***^ (-0.20 – -0.15) | -0.16 ^***^ (-0.19 – -0.14) | -0.16 ^***^ (-0.19 – -0.13) | |
| Number of chronic diseases | -0.01 ^***^ (-0.02 – -0.01) | -0.02 ^***^ (-0.02 – -0.01) | -0.01 ^***^ (-0.02 – -0.01) | |
| Number of depressive symptoms | -0.03 ^***^ (-0.03 – -0.03) | -0.03 ^***^ (-0.03 – -0.02) | -0.03 ^***^ (-0.03 – -0.02) | |
| IEO | -0.23  (-0.88 – 0.41) | -0.39  (-1.11 – 0.33) | -1.73 ^**^ (-2.76 – -0.71) | |
| IEO*female | -1.17 ^***^ (-1.41 – -0.93) | -0.94 ^***^ (-1.20 – -0.69) | -0.61 ^***^ (-0.87 – -0.35) | |
| IEO*ISCED 3 | -0.15  (-0.41 – 0.12) | -0.27  (-0.55 – 0.01) | -0.38 ^**^ (-0.66 – -0.10) | |
| IEO*ISCED 4-6 | -0.26  (-0.54 – 0.01) | -0.38 ^*^ (-0.67 – -0.08) | -0.35 ^*^ (-0.64 – -0.05) | |
| ISCED 3*female | 0.08 ^***^ (0.05 – 0.11) | 0.10 ^***^ (0.07 – 0.13) | 0.10 ^***^ (0.06 – 0.13) | |
| ISCED 4-6*female | 0.05 ^**^ (0.02 – 0.09) | 0.09 ^***^ (0.06 – 0.13) | 0.13 ^***^ (0.09 – 0.16) | |
| IEO*ISCED 3*female | 0.80 ^***^ (0.46 – 1.14) | 0.60 ^**^ (0.24 – 0.96) | 0.83 ^***^ (0.47 – 1.20) | |
| IEO*ISCED 4-6*female | 0.83 ^***^ (0.47 – 1.19) | 0.68 ^***^ (0.30 – 1.06) | 0.54 ^**^ (0.16 – 0.93) | |
| **Random Effects** | | | | |
| σ^2^ | 0.56 | 0.55 | 0.43 | |
| τ_00 individual-level_ | 0.26 | 0.32 | 0.34 | |
| τ_00 country-cohort_ | 0.04 | 0.05 | 0.10 | |
| ICC | 0.35 | 0.40 | 0.50 | |
| N | 46,448 | 46,447 | 46,438 | |
| Number of country-cohorts | 49 | 49 | 49 | |
| Observations | 161,168 | 161,273 | 139,091 | |
| Marginal R^2^ / Conditional R^2^ | 0.123 / 0.427 | 0.120 / 0.471 | 0.146 / 0.577 | |
| Note. ** p*<0.05.*** p*<0.01*. *** p*<0.001. std., standardized, CI, confidence interval, ISCED, International Standard Classification of Education, No., number, IEO, Inequality of educational opportunity, σ^2^ , mean random effect variance (overall), τ_00_, random effect (between-units) variances on individual level and country-cohort level, ICC, Intra-class correlation, N, number of respondents, Marginal R^2^, variance explained by fixed effects, Conditional R^2^, variance explained by fixed and random effects. Adjusted for age, age squared, indicator of first testing, current job situation, cohabitation status. | | | | |
